# A GTPV-Based Murine Model Recapitulating Key Features of Lumpy Skin Disease for Preclinical Vaccine Evaluation

**DOI:** 10.3390/ani16040611

**Published:** 2026-02-14

**Authors:** Wanfeng Ji, Xinjun Zhou, Sen Zhang, Xinwei Yuan, Wenying Wu, Xiaowen Xu, Aizhen Guo, Yingyu Chen

**Affiliations:** 1 National Key Laboratory of Agricultural Microbiology, Hubei Hongshan Laboratory, College of Veterinary Medicine, Huazhong Agricultural University, Wuhan 430070, China; wanfeng0609@webmail.hzau.edu.cn (W.J.); 2023302120148@webmail.hzau.edu.cn (X.Z.); zhangs416@webmail.hzau.edu.cn (S.Z.); yxw9766@webmail.hzau.edu.cn (X.Y.); wuwenying@webmail.hzau.edu.cn (W.W.); xiaowenxu@webmail.hzau.edu.cn (X.X.); aizhen@mail.hzau.edu.cn (A.G.); 2 Hubei International Scientific and Technological Cooperation Base of Veterinary Epidemiology, The Cooperative Innovation Center for Sustainable Pig Production, Wuhan 430070, China; 3 Key Laboratory of Development of Veterinary Diagnostic Products, Ministry of Agriculture and Rural Affair, Wuhan 430070, China; 4 Hubei Jiangxia Laboratory, Wuhan 430200, China

**Keywords:** goatpox virus, lumpy skin disease virus, murine model

## Abstract

The global spread of lumpy skin disease (LSD), a devastating cattle disease caused by the lumpy skin disease virus (LSDV), is hampered by the lack of a practical small animal model for vaccine evaluation. Here, we established a mouse model by challenging C57BL/6 mice with goatpox virus (GTPV), a close relative of LSDV. Intranasal inoculation with a high dose (10^4.5^ TCID_50_) of GTPV induced robust infection characterized by weight loss, fever, high viral loads in the lungs and livers, and significant pulmonary pathology including immune cell infiltration and alveolar wall thickening. Infected mice mounted potent humoral immunity, with rapid IgM and sustained IgG and GTPV-specific binding antibody responses. The infection also elicited a dynamic cellular immune response, marked by biphasic IFN-γ production and dose-dependent increases in IL-17A and TNF-α. This GTPV-based murine model effectively recapitulates critical aspects of LSDV pathogenesis and immunity, providing a valuable and accessible tool for the accelerated development and preclinical assessment of novel LSDV vaccines.

## Simple Summary

Lumpy skin disease poses a major threat to cattle farming, causing severe sickness and financial loss globally. A major obstacle to stopping this virus is that using cattle for every experiment is too expensive and complex, yet there have been no suitable lab animals to use instead. To address this, our research created a new way to study the disease using mice. We used a closely related goat virus as a substitute to mimic the infection. We found that delivering this virus into the nose caused the mice to lose weight and develop lung inflammation, closely mirroring the natural disease process. Importantly, the mice produced a strong immune response similar to what is seen in cattle. This confirms that our mouse model is a reliable tool for initial testing. It offers scientists a faster, cheaper method to screen potential vaccines before moving to livestock trials.

## 1. Introduction

Lumpy skin disease (LSD), caused by the lumpy skin disease virus (LSDV), presents a significant economic threat to the global cattle industry [1,2]. The disease is characterized by fever, cutaneous nodules, and reduced productivity, resulting in substantial direct economic losses [3]. Beyond clinical severity, LSD imposes profound indirect costs due to trade restrictions and outbreak containment measures [4,5]. Historically confined to Africa, the virus has recently expanded across the Middle East, Europe, and Asia [6,7,8], including a 2019 incursion into China that highlighted its rapid transboundary spread [9]. This spread is facilitated primarily by mechanical transmission through biting insect vectors, such as stable flies and mosquitoes, which are considered major drivers of field epidemics [10,11].

Vaccination remains the primary strategy for controlling capripoxvirus infections [12]. However, current control efforts are hindered by the lack of specific, commercially available, and highly effective LSDV vaccine [13]. While heterologous vaccines based on sheeppox or goatpox viruses (GTPV) provide cross-protection, their efficacy is often variable, creating an urgent need for next-generation LSDV vaccines [14,15]. A critical bottleneck in developing these vaccines is the scarcity of practical small animal models for preliminary screening [12]. Cattle studies are expensive, logistically complex, and pose significant practical and regulatory challenges. Previous attempts to establish LSDV infection in mice have been limited by inconsistent skin lesion formation and a lack of comprehensive virological indicators, largely because LSDV does not replicate efficiently in murine hosts [16,17]. Recent studies have explored various small-animal models for LSDV; for instance, hamsters and guinea pigs have been shown to support more productive viral replication and even develop pathognomonic skin nodules [17]. However, these models often face challenges such as limited availability of specialized immunological reagents, higher costs, and greater genetic heterogeneity. In contrast, murine models, though often lacking certain clinical signs like nodules, provide a highly standardized and genetically uniform platform. Leveraging the extensive immunological toolkit available for mice allows for a deeper and more scalable analysis of vaccine-induced protection, which is essential for early-stage vaccine de-risking. Building on established methodologies from orthopoxvirus research [18,19,20], we reasoned that mice could provide a standardized platform for the comparative evaluation of LSDV vaccine candidates based on robust immune readouts.

To overcome these limitations, we utilized the closely related GTPV as a surrogate pathogen. GTPV shares over >96% nucleotide sequence identity with LSDV and is phylogenetically closer to it than sheeppox virus [21]. In this study, we employed the attenuated GTPV AV41 strain. This strain was originally derived from the virulent GTPV-AV40 field isolate (Qinghai province, China, 1959). It was created as a vaccine strain via continuous passage in goat and sheep testis cells at 30 °C in 1985 [22]. Although the precise molecular determinants of its attenuation are not fully elucidated, this empirical attenuation process is well-established. Notably, the AV41 strain has been evaluated in cattle and shown to induce cross-protective immune responses against LSDV [23]. Therefore, AV41 represents a relevant and practical immunogen for modeling cross-protective immunity in a murine system. Despite this potential, a robust, immunologically characterized murine model for capripoxviruses has not yet been fully validated for vaccine research.

This study aims to develop and characterize a murine infection model using the attenuated GTPV AV41 strain. We systematically compared intranasal and subcutaneous inoculation routes to identify the most effective method for establishing infection. Subsequently, we investigated the dose-dependent effects of intranasal challenge on the clinical progression, viral tropism, pathology, and immune dynamics. We demonstrate that intranasal infection of C57BL/6 mice with GTPV recapitulates key host–pathogen interactions, providing an accessible and informative platform for the preclinical evaluation of LSDV vaccines.

## 2. Materials and Methods

### 2.1. Viruses, Animals and Ethics

GTPV AV41 strain (GenBank NO: MH381810.1) was obtained commercially. This cell culture-adapted vaccine strain was propagated and titrated on primary lamb testis cells to determine the 50% tissue culture infectious dose (TCID_50_). Although attenuated, the AV41 strain retains the core antigenic properties of wild-type GTPV, making it a suitable and biosafe surrogate for modeling infection and immune responses.

Specific pathogen-free female C57BL/6 mice (6–8 weeks old) were housed under standard laboratory conditions. All animal procedures were conducted in strict accordance with institutional guidelines and approved by the Animal Experiment Ethics Committee of Huazhong Agricultural University (Approval Number: HZAUMO-2024-0125).

### 2.2. Experimental Design and Infection Model

To establish a murine model for LSDV research, we first determined the optimal route of infection (Table 1, Experiment 1). Mice were lightly anesthetized with isoflurane and challenged either intranasal (IN) or subcutaneously (SC) with 100 μL of GTPV at a dose of 10^3.5^TCID_50_ (*n* = 3 per group). Control mice (*n* = 6) received an equal volume of DMEM via the same routes, and their data were pooled for analysis as no route-specific effects were observed in controls.

Based on the finding that intranasal inoculation induced superior systemic infection, we proceeded to characterize the dose-dependent responses (Table 1, Experiment 2). A separate cohort of mice was challenged intranasally with a high dose (10^4.5^TCID_50_, *n* = 12) or a low dose (10^3.5^TCID_50_, *n* = 12) of GTPV, alongside DMEM-treated controls (*n* = 6). The mice were lightly anesthetized using isoflurane. Once the pedal reflex was absent but spontaneous breathing remained regular, each mouse was held in an upright position. Using a micropipette (Eppendorf SE, Hamburg, Germany), a total volume of 100 μL of the virus inoculum (or DMEM for controls) was slowly and alternately administered onto the nares of the mouse, allowing the animal to inhale the droplet naturally between breaths.

### 2.3. Clinical and Virological Monitoring

Mice were monitored daily for changes in body weight and rectal temperature. To quantify viral replication kinetics, viral DNA loads in whole blood and homogenized tissues (hearts, livers, spleens, lungs, and kidneys) were determined at specified time points by a previously established qPCR assay [24]. Briefly, approximately 0.1 g of each tissue snippet was weighed and homogenized. Total DNA was then extracted from all tissue homogenates and whole blood samples using the Virus DNA/RNA Extraction Kit 2.0 (Prepackaged) (Nanjing Vazyme Co., Ltd., Nanjing, China), and a TaqMan assay targeting the CaPV-074 gene was performed using the primers and probe listed in Table 2. Standard curves generated from a cloned plasmid template were used for absolute quantification, with results expressed as viral copy numbers per microliter of blood or per milligram of tissue.

### 2.4. Assessment of Immune Responses

Humoral immunity was profiled by monitoring systemic antibody kinetics, with a specific focus on virus-specific binding antibodies serum samples were collected weekly and analyzed for total IgG, total IgM, and GTPV-specific binding antibodies. Total IgG and IgM levels were quantified using commercial murine ELISA kits (Jiangsu Meimian Industrial Co., Ltd., Yancheng, China) in accordance with the manufacturer’s protocols.

GTPV-specific binding antibodies were detected using a validated in-house indirect ELISA. Optimization of the inactivated viral antigen coating concentration and serum dilution factor were performed via checkboard titration. Briefly, microplates were coated with the optimized viral antigen, blocked, and incubated with serial dilutions of mouse serum. Bound antibodies were visualized using a horseradish peroxidase (HRP)-conjugated goat anti-mouse IgG secondary antibody (Abbkine Scientific Co., Ltd., Beijing, China) followed by TMB substrate development. Absorbance was measured at 450 nm.

Cellular immune responses were evaluated by quantifying key serum cytokines. Concentrations of IL-4, IL-17A, IFN-γ, and TNF-α were determined at 4, 8, and 12 days post-challenge (dpc) using corresponding commercial ELISA kits (Jiangsu Meimian Industrial Co., Ltd., Yancheng, China), following the manufacturer’s instructions.

### 2.5. Histopathological and Immunohistochemical Analysis

Tissues (hearts, livers, spleens, lungs, and kidneys) were collected for pathological examination. Organs were fixed in 4% paraformaldehyde, embedded in paraffin, sectioned, and stained with hematoxylin and eosin (H&E). Given the predominant pathology observed in the lungs, a detailed analysis was performed on this organ. Lung injury was quantified using a validated scoring system [25]. Furthermore, to visualize viral antigen distribution, lung sections were subjected to immunohistochemistry (IHC) using LSDV-positive bovine serum as the primary antibody, followed by an HRP-conjugated secondary antibody and DAB chromogen development.

### 2.6. Statistical Analysis

All data are presented as the mean ± standard error of the mean (SEM). Statistical analyses were performed using GraphPad Prism version 9.5.1. Differences between two groups were assessed by an unpaired Student’s *t*-test. For comparisons across multiple groups or at multiple time points, one-way or two-way analysis of variance (ANOVA) was applied, respectively. A *p*-value of less than 0.05 was considered statistically significant. The following *p*-value thresholds were used to determine the statistical significance: *p* < 0.05 (*), *p* < 0.01 (**), *p*  <  0.001 (***) and *p*  <  0.0001 (****).

## 3. Results

### 3.1. Intranasal Inoculation Outperforms Subcutaneous Route in Establishing a Disseminated Infection

**Figure 1:**
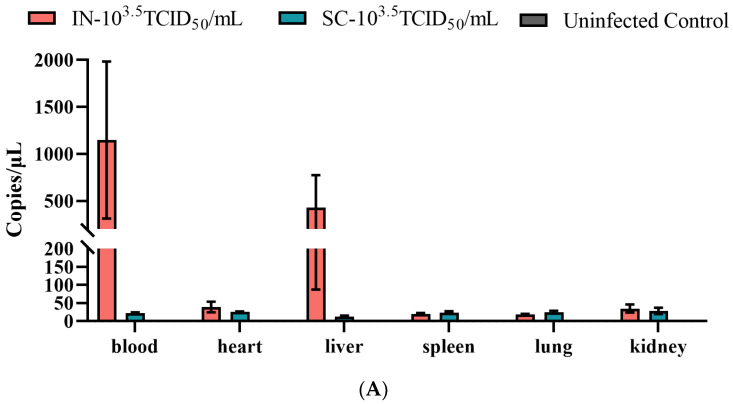

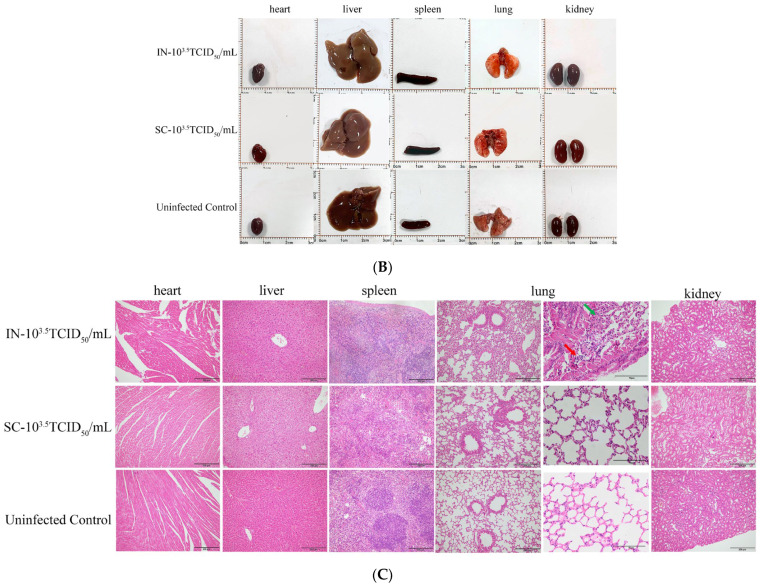
Intranasal inoculation of GTPV leads to enhanced systemic viral dissemination and pathology. C57BL/6 mice were inoculated intranasally (IN) or subcutaneously (SC) with 10^3^·^5^ TCID_50_ GTPV (*n* = 3 per group). Control mice received DMEM. Viral DNA copies were quantified by qPCR. (**A**) Viral loads in blood and organs at 14 days post-challenge (dpc). (**B**) Representative gross pathology of organs at 14 dpc. (**C**) Representative histopathological images of H&E-stained organ sections. For the lung, higher-magnification insets are shown with arrows indicating alveolar epithelial hyperplasia (green) and immune cell infiltration (red). Scale bars: 200 μm (main panels), 50 μm (higher-magnification).

To establish a murine model for LSDV, we first evaluated the efficacy of two challenge routes. Mice inoculated intranasally with 10^3.5^ TCID_50_ GTPV showed no overt clinical signs but developed a substantially higher viral burden compared to the subcutaneously challenged group. At 14 dpc, qPCR analysis revealed that the IN group had markedly elevated viral DNA copies in the blood, and most notably, in the liver, where levels exceeded 500 copies/μL, suggesting a specific tropism (Figure 1A). In contrast, viral loads in the SC group remained low across all tissues examined.

Histopathological assessment aligned with the virological data. While gross pathology was unremarkable aside from lighter liver coloration in challenged groups (Figure 1B), microscopic analysis uncovered distinct lesions. The IN group exhibited hepatocyte swelling, renal tubular epithelial cell vacuolation, and splenic architectural disruption. In the lung, higher-magnification images demonstrated alveolar epithelial hyperplasia (green arrow) and immune cell infiltration (red arrow). These changes were minimal or absent in the SC and control groups (Figure 1C). Consequently, the intranasal route was selected for further model characterization.

### 3.2. Infection Dose Dictates Clinical Severity, Viral Kinetics, and Pulmonary Pathology

**Figure 2:**
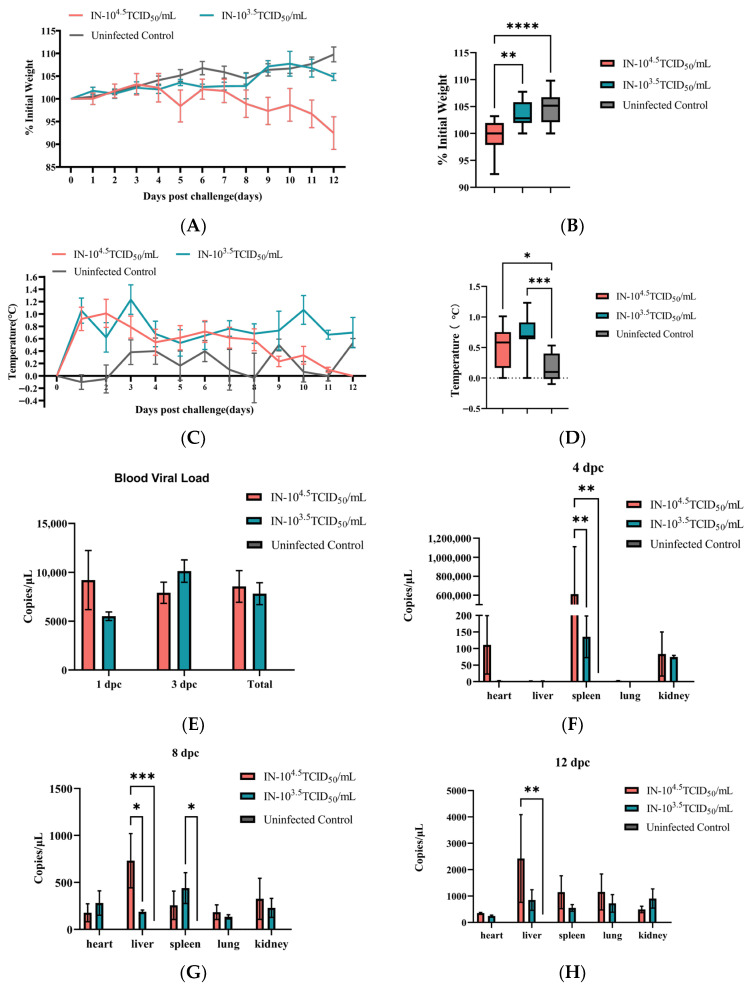
Clinical phenotypes in C57BL/6 mice after GTPV infection. (**A**) Daily body weight change over time. (**B**) Total body weight change summarized across the entire observation period. (**C**) Daily rectal temperature over time. (**D**) Total temperature change summarized across the entire observation period. Blood viral load (**E**) and organ viral load (**F**–**H**) in C57BL/6 mice following intranasal challenged with 100 µL 10^4.5^TCID_50_/mL or 10^3.5^TCID_50_/mL GTPV, compared to uninfected mice. * *p* < 0.05, ** *p* < 0.01, *** *p* < 0.001, **** *p* < 0.0001 vs. control group.

We next investigated the impact of viral inoculum size using two intranasal doses. Mice receiving the high dose (10^4.5^ TCID_50_) experienced significant weight loss, declined to its lowest level below baseline by 12 dpc, and a rapid febrile response that peaked early and then normalized (Figure 2A–D). The low-dose (10^3.5^ TCID_50_) group, in contrast, maintained a moderate weight gain and a sustained, low-grade elevation in temperature throughout the observation period.

Virological profiling demonstrated a clear dose-dependent response. The high-dose group initiated with a blood viral load exceeding 10^4^ copies/μL, which was significantly higher than controls at 1 dpc (Figure 2E). This high viral burden was maintained in organs, with the spleen harboring over 10^6^ copies by 4 dpc and the liver showing significantly elevated loads at 8 and 12 dpc, starkly contrasting with the more modest replication seen in the low-dose group (Figure 2F–H).

**Figure 3:**
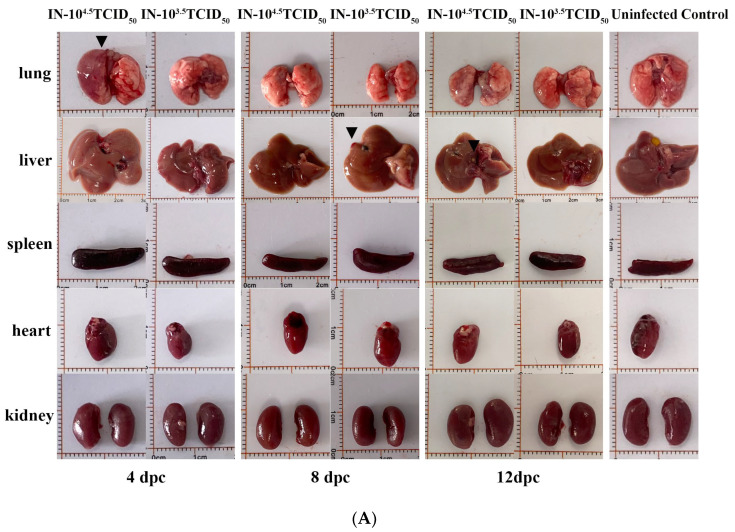

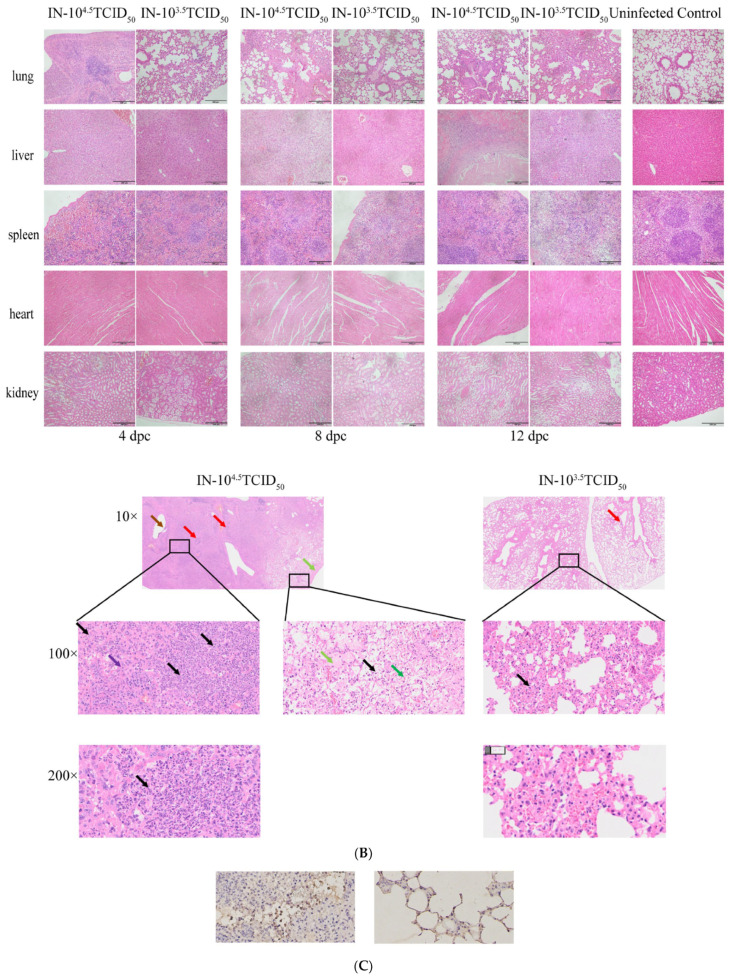

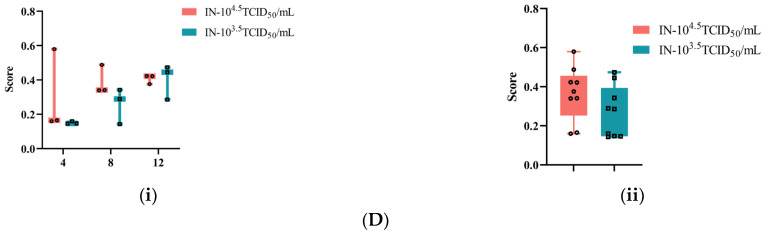
Dose-dependent pathological progression in the lungs following GTPV challenge. (**A**) Representative gross images of livers and lungs at 4, 8, and 12 dpc. Black triangles indicate nodular hepatic lesions and pulmonary consolidation. (**B**) Representative histopathology of lung tissues (H&E staining) at 4 dpc. The lower panels show magnified views of the boxed areas. Arrows indicate: black, neutrophils/macrophages; red, lymphocyte infiltration; brown, bronchial inflammation; purple, alveolar wall thickening/connective tissue proliferation; light green, pulmonary edema; green, mild hemorrhage. (**C**) Immunohistochemical (IHC) detection of viral antigens (brown) in lung tissues (200×). (**D**) (**i**) Quantitative lung injury scores over time (4, 8, and 12 dpc) and (**ii**) the overall score for the entire challenge period.

Pathological manifestations reinforced the dose–severity relationship. Gross examination at 12 dpc identified nodular hepatic lesions and substantial lung consolidation at 4 dpc in the high-dose group (Figure 3A). Histologically, the high-dose challenge induced severe pulmonary pathology by 4 dpc, characterized by massive infiltration of neutrophils and macrophages into alveoli, inflammatory cell bronchial infiltration, mild alveolar wall thickening, and evidence of edema and hemorrhage (Figure 3B). The low-dose group presented with milder, focal neutrophilic infiltration and alveolar constriction. Immunohistochemistry confirmed the presence of viral antigens within alveolar septal cells in infected lungs (Figure 3C). Quantitative lung injury scoring confirmed that the high dose caused rapidly severe damage, while the low dose led to a progressively worsening pathology over time (Figure 3D).

### 3.3. A Dynamic Humoral and Cellular Immune Landscape Is Revealed in a Dose- and Time-Dependent Manner

**Figure 4:**
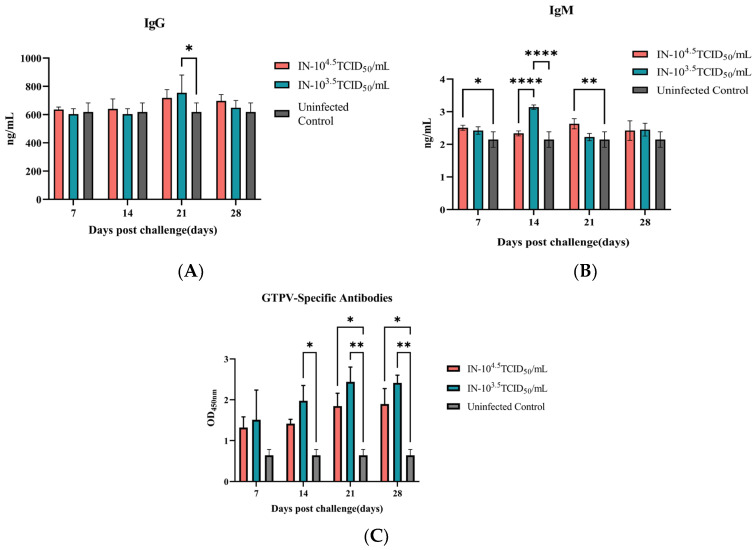
GTPV infection elicits a dynamic and dose-dependent humoral immune response. Serum levels of (**A**) total IgG, (**B**) GTPV-specific binding antibodies, and (**C**) total IgM were measured by ELISA in mice intranasally challenged with high-dose (10^4.5^ TCID_50_), low-dose (10^3.5^ TCID_50_) GTPV, or DMEM control over 28 days. Data are presented as mean ± SEM (*n* = 3 per group per time point). * *p* < 0.05, ** *p* < 0.01, **** *p* < 0.0001 vs. control group.

The immunological response to GTPV infection was multifaceted. Humorally, total IgG levels rose progressively, peaking at 21 dpc, with the low-dose group showing a statistically significant increase over uninfected controls (Figure 4A). Serum IgM exhibited a more complex kinetic: the high-dose group mounted an early, significant rise by 7 dpc, whereas the low-dose group displayed a delayed but sharp peak at 14 dpc, which was significantly higher than both the high-dose and control groups at that timepoint (Figure 4B). Critically, GTPV-specific binding antibodies were detectable in both challenged groups, with the low-dose group consistently showing an earlier and stronger response from 14 to 28 dpc (Figure 4C).

**Figure 5:**
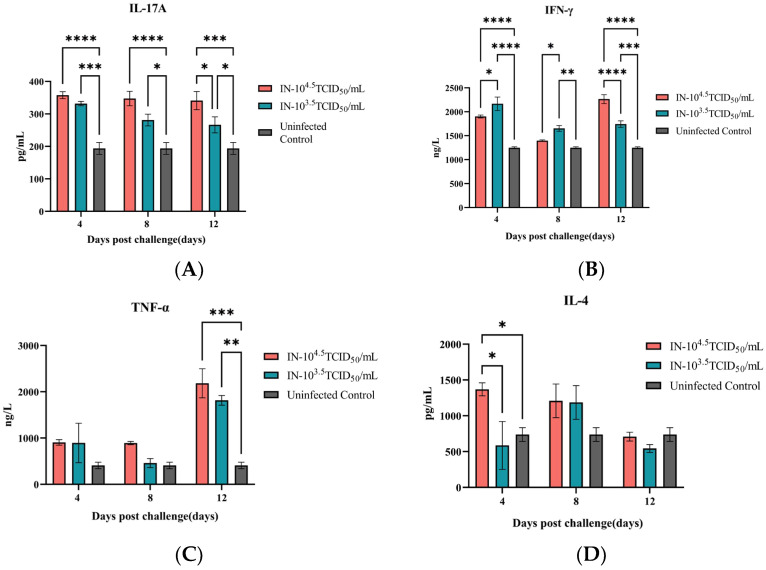
Distinct cytokine profiles reveal a biphasic and Th17-skewed cellular immune response. Serum concentrations of IL-17A (**A**), IFN-γ (**B**), TNF-α (**C**) and IL-4 (**D**) were quantified by ELISA at 4, 8, and 12 dpc in mice challenged intranasally with high-dose or low-dose GTPV, alongside uninfected controls. Data are shown as mean ± SEM. * *p* < 0.05, ** *p* < 0.01, *** *p* < 0.001, **** *p* < 0.0001.

The cellular immune response was defined by distinct cytokine patterns. IL-17A was significantly upregulated in both challenged groups at all time points, with a clear dose-dependency evident at 12 dpc (Figure 5A). IFN-γ production followed a biphasic pattern in the high-dose group; it was initially suppressed compared to the low-dose group at 4 and 8 dpc, but subsequently surged to become the highest among all groups by 12 dpc (Figure 5B). TNF-α levels remained low initially but saw a substantial, dose-dependent increase by 12 dpc, with both challenged groups significantly exceeding the control (Figure 5C). In contrast, IL-4 was only transiently elevated in the high-dose group at 4 dpc, indicating a limited and early Th2 involvement (Figure 5D).

## 4. Discussion

The critical barrier to developing effective vaccines against lumpy skin disease has been the absence of a practical and immunologically informative small animal model. In this study, we successfully bridged this gap by establishing and characterizing a murine model using a closely related capripoxvirus, GTPV. Our data demonstrate that intranasal inoculation of C57BL/6 mice with GTPV results in a productive, disseminated infection. The value of this model stems from its ability to mirror functional aspects of systemic capripoxvirus infection, rather than from genetic similarity alone. In mice, infection results in high viral loads in the liver, spleen, and blood, together with marked lung pathology including immune infiltration and alveolar damage. This combination of viremia, visceral infection, and pneumonia directly parallels the established pathogenesis of natural LSDV in cattle, which is defined by systemic spread to visceral and lymphoid tissues and frequent broncho-interstitial pneumonia [26]. By reproducing these key facets of early infection—the pattern and process of infection—the model offers a physiologically relevant system for preclinical vaccine assessment.

### 4.1. The Murine Model: Rational Route Selection and Pathological Relevance

Our systematic comparison revealed that the intranasal route was markedly superior to the subcutaneous route in establishing a systemic infection, as evidenced by significantly higher viral loads in multiple organs, particularly the liver. This finding aligns with established models for other poxviruses, where respiratory exposure often leads to broader systemic dissemination [18]. The pronounced hepatic tropism observed is a noteworthy finding, as liver involvement, though not the primary clinical sign, has been documented in natural capripoxvirus infections in ruminants [24,27,28]. This suggests that our model captures an underlying aspect of viral pathogenesis that may be crucial for understanding systemic spread. The subsequent pulmonary pathology—characterized by immune cell infiltration, alveolar damage, and the presence of viral antigen in alveolar cells—further validates the model’s relevance, mirroring respiratory complications observed in severe cases.

### 4.2. Dose-Dependent Dynamics: Decoupling Clinical Presentation from Immune Activation

A pivotal finding of this study is the inverse relationship between the viral inoculum dose and the host’s adaptive immunological response. While the high-dose challenge induced acute disease characterized by weight loss, fever, and severe pathology, the low-dose challenge—despite its milder clinical presentation—elicited a significantly more robust and rapid GTPV-specific binding antibody response (Figure 4C). This observation suggests that a lower antigenic load may optimize the adaptive humoral response by maintaining a critical balance between immunogenicity and immunopathology [29,30].

We hypothesize that the overwhelming viral replication and associated tissue damage in the high-dose group may disrupt the coordinated development of the adaptive immune response. Excessive antigen load and systemic inflammation can disrupt antigen presentation, induce T-cell exhaustion, or skew the immune profile toward innate pathways at the expense of specific antibody production. In contrast, the controlled infection dynamics in the low-dose group likely facilitate more efficient lymphocyte priming and germinal center activity, ultimately yielding a stronger and more specific humoral response.

From a translational perspective, these findings underscore a critical consideration for vaccine challenge models: the most severe clinical phenotype does not necessarily yield the most informative immunological readout. A key strength of this GTPV-mouse system is its ability to distinguish between pathogenicity and immunogenicity. While reducing the challenge dose leads to attenuated clinical signs and a concurrently robust adaptive immune response in our murine model, this observation aligns with the biological principles of dose-dependency often encountered in natural hosts. In cattle, although LSDV infection typically manifests with varying severity based on viral load, the prohibitive cost and ethical complexity of bovine trials make it difficult to precisely define the threshold between subclinical immunogenicity and systemic disease. By establishing this murine platform, we provide a high-resolution tool to map these dose–response dynamics. This serves as a critical de-risking strategy for future cattle trials, allowing for the identification of optimal challenge doses that can distinguish between partial and full vaccine-induced protection, rather than focusing solely on lethal clinical endpoints. For preclinical vaccine evaluation, this model allows researchers to select a challenge dose that optimizes the detection of vaccine-induced immune correlates of protection.

### 4.3. Contextualizing Our Model Within the Evolving Research Landscape

Our findings present a complementary yet distinct perspective to the recent murine model described by Xie et al. [16], which utilized a high-dose (10^7^ PFU) intradermal challenge with wild-type LSDV to study skin pathogenesis and a specific immune evasion gene (LSDV012), and reported skin nodules as a primary endpoint. The absence of cutaneous lesions in our GTPV model underscores the phenotypic variability that can exist between different capripoxvirus–isolate–host combinations. Importantly, our model demonstrates that a productive and informative infection capable of stimulating comprehensive humoral and cellular immunity can be achieved and monitored through systemic virological and immunological parameters, even in the absence of the classic skin phenotype. This expands the toolbox for researchers, offering an alternative model particularly suited for studying systemic pathogenesis and immunity, which are the ultimate targets of vaccine-induced protection.

### 4.4. Limitations and Future Directions

We acknowledge that this model, which relies on a surrogate virus, does not fully replicate the pathogenesis of LSDV in cattle. Most notably, the murine model lacks the cutaneous nodules characteristic of the natural disease. We therefore clarify that the choice of mice was a strategic decision for this early-stage immune profiling, accepting this known limitation to prioritize a controlled and efficient evaluation of immune responses before more complex animal trials. While cutaneous nodules are not reproduced, the model’s strength lies in its ability to simulate systemic viremia and organ tropism. In the context of vaccine development, the prevention of systemic viral spread to visceral organs and the lungs is a primary goal. Therefore, a model that focuses on visceral and respiratory protection provides a robust and high-resolution platform for screening candidates before moving to costly bovine trials. The selection of mice over other potential hosts like hamsters or guinea pigs—which may more closely mimic the cutaneous pathology of LSD [17]—was informed by the need for a high-throughput, immunologically traceable system. The precision offered by murine-specific reagents and the ability to utilize standardized infection protocols facilitate the identification of subtle immune correlates of protection that might be obscured in less-characterized animal systems. Thus, our model complements existing pathological models by focusing on the systemic and molecular aspects of vaccine efficacy. However, the extensive genomic and antigenic homology between GTPV and LSDV supports the model’s utility for preliminary vaccine screening.

Despite the induction of robust binding antibodies against the whole GTPV virion, our in vitro neutralization assays did not detect significant neutralizing activity. This apparent discrepancy is well-documented in poxvirus immunology. Antibodies elicited against the intact virion constitute a heterogeneous pool, targeting both surface-exposed and internal viral proteins. As highlighted by Vernuccio [31], many immunodominant antigens targeted by such serum are not surface-exposed and do not contribute to neutralization. Furthermore, many antibodies, while detectable in binding assays, may not recognize the specific conformational epitopes on key viral surface proteins that are critical for blocking viral entry. Importantly, protective immunity against poxviruses is multifaceted. It can be mediated not only by classical neutralizing antibodies but also by antibody effector function and potent cellular immune responses [32]. Therefore, our murine challenge model likely captures these complementary protective mechanisms in vivo, providing a more holistic assessment of vaccine-induced protection than in vitro neutralization alone. While the high levels of binding antibodies indicate successful antigen recognition and B-cell activation, neutralizing antibodies are the primary correlate of protective immunity. To build upon these findings, future studies will address these gaps by utilizing this model to evaluate candidate LSDV vaccines and establishing correlations between immune parameters—including functional neutralization titers and IFN-γ levels—and protection against challenge. Finally, the pronounced hepatic tropism observed in this model warrants further investigation into the molecular mechanisms governing capripoxvirus pathogenesis.

## 5. Conclusions

In summary, we have developed a robust and reproducible murine model for LSDV research using GTPV. By establishing the intranasal route as the most effective method and delineating the dose-dependent clinical, pathological, and immunological landscape of the infection, we provide a validated and accessible platform. This model is poised to significantly accelerate the preliminary screening and mechanistic evaluation of novel vaccines and therapeutics, thereby contributing to the global effort to control the spread of this economically devastating disease.

## Figures and Tables

**Table 1:** Summary of experimental design.

Experiment	Group Name	Route	Dose	*n*	Purpose
1 (Route)	IN-10^3.5^	Intranasal (IN)	10^3.5^ TCID_50_	3	Compare infection routes
	SC-10^3.5^	Subcutaneous (SC)	10^3.5^ TCID_50_	3	Compare infection routes
	Control	IN/SC	DMEM	6 (pooled ^[1]^)	Vehicle control
2 (Dose)	IN-10^4.5^	Intranasal (IN)	10^4.5^ TCID_50_	12	Evaluate dose–response
	IN-10^3.5^	Intranasal (IN)	10^3.5^ TCID_50_	12	Evaluate dose–response
	Control	IN	DMEM	6	Vehicle control

^[1]^ a Data from control mice receiving DMEM via intranasal (IN) or subcutaneous (SC) routes were pooled for analysis because no statistically significant differences were observed between these two control groups in any of the measured outcomes.

**Table 2:** qPCR Primer and probe sequences.

Item	Sequence (5′→3′)
CaPV-074F1	AAAACGGTATATGGAATAGAGTTGGAA
CaPV-074R1	AAATGAAACCAATGGATGGGATA
TaqMan probe CaPV-074P1	6FAM-TGGCTCATAGATTTCCT-MGBNFQ

## Data Availability

The data supporting the findings of this study are available within the article. Further inquiries can be directed to the corresponding authors.
